# Clinical significance of CADM1/TSLC1/IgSF4 expression in Adult-T cell leukemia/lymphoma (ATLL): identification of various types of ATLL cells

**DOI:** 10.1186/1742-4690-8-S1-A49

**Published:** 2011-06-06

**Authors:** Shingo Nakahata, Yusuke Saito, Kosuke Marutsuka, Tomonori Hidaka, Koichi Maeda, Atae Utsunomiya, Akihiko Okayama, Yoko Kubuki, Kazuya Shimoda, Yujiro Asada, Yoshinori Ukai, Gene Kuosawa, Kazuhiro Morishita

**Affiliations:** 1 Faculty of Medicine, University of Miyazaki, Miyazaki, Japan; 2 Miyakonojo National Hospital, Miyazaki, Japan; 3 Department of Hematology, Imamura Bun-in Hospital, Kagoshima, 890-0067, Japan; 4 Division of Antibody Project, Institute for Comprehensive Medical Science, Fujita Health University, Aichi, Japan

## 

Cell adhesion molecule 1 (CADM1/TSLC1/IgSF4) was recently identified as a novel cell surface maker for adult T-cell leukemia/lymphoma (ATLL). In this manuscript, we developed several kinds of antibodies for diagnostic tools identifying CADM1-positive ATLL cells. 107 kDa of CADM1 protein was detected in the ATLL cell lines and a 72 kDa of soluble CADM1 protein was detected in serum from ATLL patients. In analysis by flow cytometry, percentages of CADM1+/CD4+ positive (DP) cells in the peripheral blood are well correlated with the percentages of CD4+/CD25+ DP cells in the peripheral bloods from various types of ATLL patients and HTLV-1 carriers. Percentages of CADM1+/CD4+ DP cell fraction were well correlated with the percentages of abnormal lymphocytes and DNA copy numbers of HTLV-1 infected or ATLL cells in the peripheral blood, particularly, with high DNA copy numbers of HTLV-1-infected lymphocytes from HTLV-1 carriers. Expression of soluble-form CADM1 is also detected in the peripheral blood from acute-type of ATLL patients with correlation of the level of soluble IL2Ra. Moreover, lymphomas derived from ATLL in paraffin-embedded tissue sections were strongly and specifically stained by CADM1 antibody, suggesting that CADM1 is one of very useful markers for identifying various types of ATLL cells.

